# Impacts of zinc caproate supplementation on growth performance, intestinal health, anti-inflammatory activity, and Zn homeostasis in weaned piglets challenged with *Escherichia coli* K88

**DOI:** 10.1186/s40104-025-01172-2

**Published:** 2025-03-14

**Authors:** Jilong Xu, Hanzhen Qiao, Liping Gan, Peng Wang, Yifeng Zhao, Zetian Lei, Yixuan Chou, Chenrui Hou, Mengqi Li, Jinrong Wang

**Affiliations:** College of Bioengineering, Henan University of Technology, Lianhua Street 100, Zhengzhou, 450001 China

**Keywords:** AGPs, ETEC K88, Pharmacological doses of ZnO, Weaned pigs, ZnCA

## Abstract

**Background:**

Enterotoxigenic *Escherichia coli* (ETEC) is one of the primary causes of diarrhea in piglets, creating substantial economic losses in the swine farming industry worldwide. This study aimed to investigate the impacts of zinc caproate (ZnCA, C_12_H_22_O_4_Zn) on the intestinal health, growth performance, inflammatory status, and Zn homeostasis of weaned piglets challenged with ETEC K88. In total, 48 weaned piglets (Duroc × Landrace × Yorkshire, 7.78 ± 0.19 kg, 28 d) were selected for a 21-d experiment. Each experimental treatment consisted of 6 replicate pens with 2 piglets each. The treatment conditions were as follows: i) a basal diet (CON), ii) a basal diet + ETEC K88 (NC), iii) a basal diet + 2,500 mg/kg of Zn (provided as zinc oxide, ZnO) + ETEC K88 (PC), and iv) a basal diet + 1,600 mg/kg of Zn (provided as ZnCA) + ETEC K88 (ZnCA).

**Results:**

The addition of 1,600 mg/kg ZnCA to the diet of post-weaning piglets effectively enhanced growth performance and nutrient digestibility and reduced the incidence of diarrhea and inflammatory reactions caused by ETEC K88 infection. These therapeutic effects were comparable to those of pharmacological doses of ZnO. In terms of improving intestinal health and Zn homeostasis in post-weaning piglets challenged with ETEC K88, the effectiveness of 1,600 mg/kg ZnCA surpassed that of pharmacological doses of ZnO.

**Conclusions:**

Overall, under the experimental conditions of this study, ZnCA exhibited the potential to reduce the pharmacological dosage of ZnO while improving intestinal health and Zn homeostasis in weaned piglets.

**Graphical Abstract:**

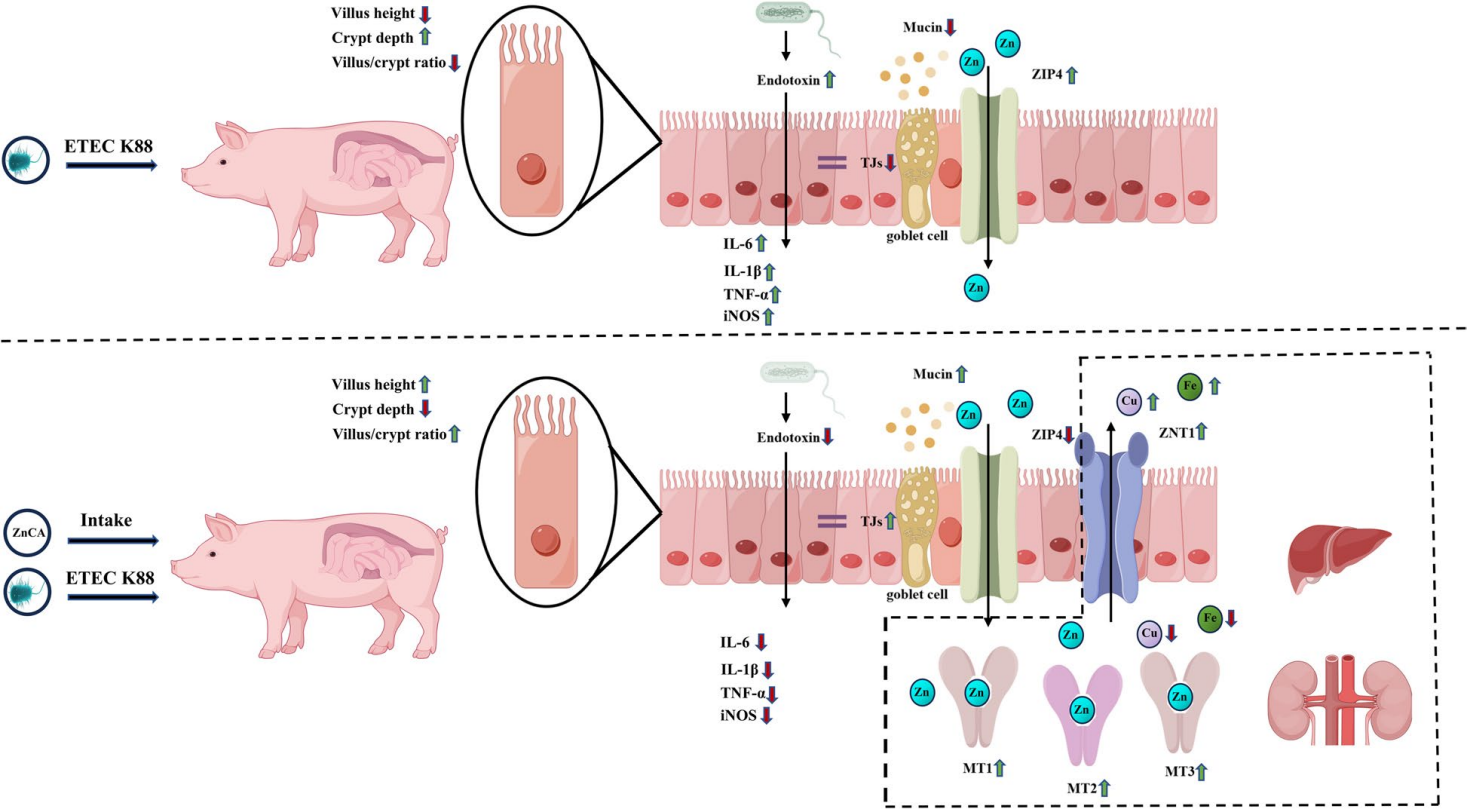

**Supplementary Information:**

The online version contains supplementary material available at 10.1186/s40104-025-01172-2.

## Background

Post-weaning diarrhea (PWD) frequently occurs in weaned piglets and is primarily the result of incomplete intestinal development and suboptimal feeding conditions on farms. This condition can become fatal, causing significant financial losses in the swine industry [1]. Enterotoxigenic *Escherichia coli* (ETEC) is the leading pathogenic cause of post-weaning colibacillosis, a common type of PWD. Globally, the predominant strain associated with PWD in piglets is ETEC K88, which typically infects these animals through the oral route [2]. The initial stages of ETEC infection involve the colonization of the intestinal epithelium and the secretion of enterotoxins. ETEC colonizes the intestinal epithelium by leveraging membrane adhesins to adhere to the intestinal mucosa and binding to glycoprotein receptors on the brush border of intestinal cells. Subsequently, it secretes enterotoxins (including heat-labile and heat-stable enterotoxins), disrupts the electrolyte balance in the intestines, and ultimately causes diarrhea [3, 4].

Historically, antibiotic growth promoters (AGPs) and pharmacological doses of zinc oxide (ZnO, 2,500 mg/kg) have been widely utilized in piglet feed to either prevent or treat PWD and enhance growth performance [5, 6]. However, serious concerns have emerged regarding the misuse of AGPs and ZnO, since these chemicals can promote bacterial resistance, lead to drug residues in animal products, and cause environmental pollution [7, 8]. Therefore, the European Union and China have imposed bans on the use of AGPs and restricted the dosage of ZnO in animal feed [9]. At present, in the European Union and China, the maximum permissible dosages of ZnO in animal feed are 150 mg/kg and 1,600 mg/kg, respectively, during the first two weeks following weaning [10].

It is becoming increasingly evident that ZnO may fail to meet the production demands of the swine farming industry in the near future [11]. Consequently, there is an urgent need to identify ZnO substitutes for this purpose. So far, a range of Zn-containing molecules with the potential to treat PWD have been studied extensively. These include inorganic Zn compounds like ZnO nanoparticles (Nano-ZnO) [12] and tetrabasic Zn chloride [13] as well as organic Zn compounds such as Zn-amino acid complexes [14], Zn-polysaccharide complexes [15], and certain coated Zn products [16, 17]. While these agents show promise in lowering the bioactive concentration of ZnO and mitigating its environmental impact, the doses employed fail to meet the European Commission’s mandate of 150 mg/kg of total Zn in complete feed [18].

Medium-chain fatty acids (MCFAs), which are promising alternatives to antibiotics, play various roles in piglet nutrition. For instance, they can enhance growth performance, boost immunity, promote the growth of beneficial intestinal microbes, and maintain intestinal homeostasis [19, 20]. MCFAs have the potential to reduce the acid-binding capacity in the intestines, thereby improving feed digestibility in weaned piglets [21]. Most MCFAs and their derivatives are generally considered safe in feed and food products and are widely used in various industries, including the daily chemicals, food, and pharmaceutical sectors [22]. Recently, studies have proposed that the synthesis of organic Zn compounds through the combination of caproic acid (CA) and Zn could potentially enhance the bioavailability of Zn, thus offering a novel avenue for effectively managing PWD and ETEC K88 infections.

In our previous study, ZnCA was successfully synthesized using a solvothermal method and demonstrated remarkable antibacterial activity against ETEC K88 in vitro [23]. Consequently, the aim of the present study was to examine the impacts of dietary supplementation with ZnCA on growth performance, inflammatory status, intestinal health, and Zn homeostasis in weaned piglets exposed to ETEC K88.

## Materials and methods

### Experimental materials

ZnO (75% Zn) was obtained from Shijiazhuang Hanying Feed Co., Ltd. (Shijiazhuang, China). Titanium dioxide (TiO_2_) was procured from Shanghai Macklin Biochemical Co., Ltd. (Shanghai, China). ZnCA (C_12_H_22_O_4_Zn), a Zn complex composed of ZnO and CA (21.91% Zn), was synthesized in our laboratory at the College of Bioengineering, Henan University of Technology.

### Animals, experimental design, and housing

Forty-eight piglets (Duroc × Landrace × Yorkshire) aged 28 d (body weight [BW] = 7.78 ± 0.19 kg) were selected for a 21-d experiment. A randomized complete design consisting of 4 treatment conditions was employed (*n* = 6 pens/treatment). Groups were matched based on BW (with weaning BW balanced across pens) and sex. Each treatment group is balanced with an equal number of males and females. Each experimental group consisted of 6 replicate pens, with 2 piglets in each pen. The control group (CON) and negative control group (NC) received a basal diet (Table 1), while the positive group (PC) received the basal diet supplemented with 2,500 mg/kg Zn (provided as ZnO). Meanwhile, the ZnCA group (ZnCA) received a basal diet supplemented with 1,600 mg/kg Zn (provided as ZnCA). All diets exceeded the nutritional recommendations proposed by the National Research Council [24]. Each pen, measuring 1.30 m × 0.5 m, was furnished with a single-side feeder and nipple drinker. The ambient temperature within the facility was initially set at 30 °C and subsequently reduced at a rate of 1.5 °C per week. The relative humidity was at 50%, and the animals had unrestricted access to feed and water throughout the 21-d study period.

**Table 1:** Composition and nutrient content of the basal diet (as-fed basis)

Ingredients, %	Content
Corn	54.55
Soybean meal (46% CP)	25
Extrusion soybean	10
Fish meal	5
Whey powder	2
Soybean oil	1
Dicalcium phosphate	0.8
Limestone	0.75
L-Lysine (98%)	0.3
NaCl	0.3
TiO_2_	0.2
Mineral premix^1^	0.08
Vitamin mix^2^	0.02
Total	100
Analyzed nutrient composition	
Gross energy, MJ/kg	19.15
Dry matter, %	88.29
Crude protein, %	22.05
Zn, mg/kg (CON and NC)	128.93
Zn, mg/kg (PC)	2,508.30
Zn, mg/kg (ZnCA)	1607.71
Calcium, %	0.80
Total phosphorus, %	0.65
Calculated nutrient composition^3^	
Metabolic energy, MJ/kg	13.31
Digestible energy, MJ/kg	14.60
L-Lysine, %	1.523
Methionine, %	0.398

^1^Premix supplied per kilogram of complete diet: Cu, 20 mg; Fe, 104 mg; Mn, 12 mg; Zn 64 mg; I, 0.8 mg; Se, 0.4 mg

^2^Premix supplied per kilogram of complete diet: Vitamin A, 6,450 IU; Vitamin D_3_, 1,520 IU; Vitamin E, 39.53 IU; Vitamin K_3_, 2 mg; Vitamin B_1_, 1.61 mg; Vitamin B_2_, 5 mg; Vitamin B_6_, 2.55 mg; Vitamin B_12_, 20 μg; D-biotin, 120 μg; Niacin, 19.6 mg; D-pantothenic acid, 12.06 mg; Folic acid, 0.99 mg; Ethoxyquin, 0.1 mg

^3^Nutrient levels were calculated according to the guidelines of the National Research Council (NRC, 2012) [24]

### ETEC K88 challenge

To guarantee that the piglets were satiated and bacterial colonization could occur effectively, feeding was halted at 21:00 on the day preceding inoculation and resumed 30 min prior to inoculation. On d 8, 9, and 10 post-weaning, piglets in the NC, PC, and ZnCA groups received 10 mL of an oral ETEC K88 suspension (3 × 10^10^ CFU/mL) twice daily (09:00 and 15:00). ETEC was obtained from the China National Center for Microbial Culture Collection (O149, K91, K88ac strains producing toxins LT, STa, and STb). Conversely, piglets in the CON group received 10 mL of normal saline orally. In order to prevent cross-contamination across the pens, partitions were installed on both sides of each enclosure while ensuring adequate ventilation. After the challenge, the frequency of enclosure disinfection was increased from once a day at 11:00 to twice a day at 09:00 and 15:00. Various disinfectants were employed to sanitize the piglets, their pens, and their surrounding environment.

### Growth performance and diarrhea scores

The BW was measured on the first and last day of the trial. Meanwhile, feed consumption was accurately recorded in each pen at 09:00 every day. Growth performance was evaluated based on the feed efficiency (G:F) using the average daily gain (ADG) and average daily feed intake (ADFI) data. Furthermore, fecal consistency was assessed daily, throughout the study, by trained personnel. These assessments were based on visual examination and followed the grading system established by Atarashi et al. [25], as follows: 0, normal feces; 1, moist or soft feces; 2, thick liquid feces or mild diarrhea; and 3, watery feces and severe diarrhea.

### Sample collection

During this study, 1 kg samples of the feed from each treatment group were individually obtained on d 0 and preserved at −20 °C. On d 19, 20, and 21, fecal samples were collected from all piglets through rectal stimulation and preserved at −20 °C for subsequent analysis. Prior to slaughter (on d 21), blood samples were collected via venipuncture from the jugular vein of one piglet per pen and placed into vacutainer coagulation tubes. A piglet whose weight was closest to the average for each treatment group was selected for blood sampling, with no consideration given to gender. All blood pecimens were allowed to clot at ambient temperature; subsequently, they were centrifuged at 3,500 × *g* and 4 °C for 15 min. The serum was collected and placed in trace element-free tubes before freezing at −80 °C for subsequent analysis. On d 21, the piglets from which blood samples had been collected were selected and euthanized. The piglets were sacrificed via jugular puncture after being anesthetized with sodium pentobarbital. The weight of the heart, liver, spleen, and kidneys was recorded after slaughter. Samples of hair, the musculus longissimus dorsi, the right lateral lobe of the liver, the jejunum, and the right kidney were obtained for Zn analysis. The tissues were washed with phosphate-buffered saline (PBS), rapidly frozen in liquid nitrogen, and preserved at −80 °C. The small intestine was separated into three distinct regions: the duodenum, jejunum, and ileum according to the method described by Hopwood et al. [26]. Tissues from the central portions of the duodenum, jejunum, and ileum were collected and washed with PBS after removing the intestinal contents. Histological samples, measuring 0.5 cm, were fixed in a 4% paraformaldehyde solution for 24 h before further morphological examination. Mucosal scrapings were gently collected from the remaining jejunal tissue using a sterile scalpel. The contents of the jejunum were also collected and immediately immersed in liquid nitrogen for preservation at −80 °C before subsequent analysis.

### Intestinal histomorphometry

Tissue samples from the duodenum, jejunum, and ileum—which had been preserved in 4% paraformaldehyde—were dehydrated, cleared, and embedded in paraffin. Following this, the samples were sliced into 4-µm sections and stained using hematoxylin and eosin (H&E). Morphological observations and measurements were carried out using a light microscope (RVL-100-G, Echo Global Logistics, Inc., California, USA) at a combined magnification of 40 × . In each section, a minimum of 12 appropriately aligned intact villi, along with the corresponding crypt depths, were identified and measured.

### Serum analyses

The enzymatic activity of alkaline phosphatase (AKP), glutamic-pyruvic transaminase (ALT/GPT), glutamic-oxaloacetic transaminase (AST/GOT), and diamine oxidase (DAO) and the levels of nitric oxide (NO) and D-lactic acid (D-LA) in the serum were measured using biochemical methods and commercial kits, strictly adhering to the manufacturer’s instructions. The levels of tumor necrosis factor-α (TNF-α), interleukin-6 (IL-6), and interleukin-1β (IL-1β) were quantified using the enzyme-linked immunosorbent assay (ELISA) technique. Additionally, a sandwich ELISA kit was employed to detect the endotoxin content in the serum. Both the biochemical reagents and ELISA kits were sourced from Nanjing Jiancheng Bioengineering Institute Co., Ltd. (Nanjing, China).

### Nutrient digestibility

Samples of feed and feces were dried at 65 °C for 72 h and then ground to a powder, which was passed through a 1-mm sieve. This processed powder was used to analyze the apparent total tract digestibility (ATTD) of nutrients in the feed. The apparent total tract digestibility (ATTD) of the nutrients—including crude protein (CP), dry matter (DM), and gross energy (GE)—was accurately assessed by utilizing the indigestible marker method, with 0.2% TiO_2_ employed as an exogenous indicator. The DM and CP content of both the feces and feed were meticulously analyzed using methods 930.15 and 990.03, respectively, outlined by the Association of Official Analytical Chemists [27]. Meanwhile, the gross energy (GE) values were determined by employing an adiabatic bomb calorimeter (Kalorimeter C6000 prozesso, IKA, Staufen, Germany). The elemental content of Zn and titanium (Ti) was determined using the AOAC method 985.0 and a spectroscope (Optima 5300 DV ICP-OES, PerkinElmer, MA, USA). The ATTD of the nutrients was then computed based on the following formula:

$$\mathrm{ATTD}\;\mathrm{of}\;\mathrm{nutrients}\;\left(\%\right)=\left\{1-\left({\mathrm{Ti}}_{\mathrm{diet}}\times{\mathrm{Nutrient}}_{\mathrm{feces}}\right)/\left({\mathrm{Ti}}_{\mathrm{feces}}\;\times\;{\mathrm{Nutrient}}_{\mathrm{diet}}\right)\right\}\times100\%$$

### Zn status

The concentrations of Zn were accurately determined in various samples, including the serum, feed, feces, hair, musculus longissimus dorsi, jejunum, liver, and kidney. Prior to analysis, all samples underwent wet digestion with a mixture of nitric acid and perchloric acid (4:1). Subsequently, the samples were diluted with ultra-pure H_2_O and examined using a spectroscope (Optima 5300 DV ICP-OES, PerkinElmer, MA, USA). The procedure involved weighing 0.5 g of lyophilized solid samples or 0.5 mL of liquid samples and then mixing them with 12 mL of nitric acid and 3 mL of perchloric acid. The samples were then digested in an adjustable electric furnace at 120 °C for 0.5 h, 180 °C for 24 h, and 220 °C until the mixtures became colorless and transparent. For Zn analysis, the liver, feed, and fecal samples were diluted with ultrapure water to reach a final volume of 25 mL. Meanwhile, the longissimus dorsi, kidney, jejunum, and hair samples were diluted to a final volume of 10 mL, and serum samples were diluted to a final volume of 5 mL.

### RNA extraction, cDNA synthesis, and real-time quantitative polymerase chain reaction (RT-qPCR)

Total RNA was isolated from 50 mg of kidney, liver, and jejunum mucosa tissues using the Freezol reagent (Vazyme, Nanjing, China). The mRNA concentrations were evaluated using a NanoDrop 2000 spectrophotometer (Thermo Fisher Scientific, Waltham, MA, USA). Complementary DNA (cDNA) was synthesized using a qPCR kit (Vazyme, Nanjing, China). RT-qPCR was conducted in a reaction volume of 20 µL, employing the SYBR qPCR Master Mix (Vazyme, Nanjing, China), on a quantitative fluorescence PCR instrument (Analytik Jena, Jena, Germany). The thermocycler protocol consisted of an initial denaturation step at 95 °C for 30 s, followed by 40 cycles of denaturation at 95 °C for 3 s and annealing/extension at 60 °C for 30 s. Melting curve analysis was conducted to assess the specificity of the amplified fragments using Dissociation Curves v1.0 software (PE Applied Biosystems). Each experimental sample was assayed using four technical replicates. The reference gene was glyceraldehyde 3-phosphate dehydrogenase (*GAPDH*), and the relative mRNA expression of the genes of interest was assessed using the 2^−^^ΔΔCt^ method. Here, the ΔCt value represents the difference between the Ct values of the target genes and the housekeeping gene. All primers, designed using sequences obtained from the National Center for Biotechnology Information (NCBI) database, are listed in Additional file 1.

### Statistical analyses

Each pen served as the experimental unit for assessing variations in growth performance, nutrient digestibility, and fecal scores among the piglets. Conversely, individual piglets were considered as the experimental units for analyzing intestinal morphology, serum parameters, and mRNA expression levels. Experimental data were analyzed with a one-way ANOVA by utilizing the Statistics Analysis System (SAS, SAS Institute, Inc., version 9.4, Cary, NC), and Tukey’s test was employed for post hoc analysis. All experimental outcomes were reported as the mean ± standard error of the mean (SEM). Significance was determined at *P* < 0.05, while a trend was noted when 0.05 < *P* < 0.1.

## Results

### Growth performance, fecal scores, and organ index

The growth performance and fecal scores of the weaned piglets are summarized in Table 2. Initially, the BW of piglets was comparable across all treatment groups. Over the 3-week period following weaning, piglets from the ZnCA group exhibited a significantly higher final BW (*P* = 0.021), ADG (*P* = 0.026), and G:F ratio (*P* = 0.026) than those from the NC group. Interestingly, there were no notable variances among the other treatment groups (*P* > 0.05). Furthermore, no obvious variations in the ADFI were observed among the treatment groups.

**Table 2:** Effects of dietary ZnCA supplementation on growth performance and fecal scores in piglets^1^

Parameters	Dietary treatments^2^				SEM^3^	*P*-value
	CON	NC	PC	ZnCA		
Initial BW, kg	7.76	7.77	7.85	7.74	0.194	0.998
Final BW, kg	14.58^b^	13.15^b^	14.92^b^	15.63^a^	0.317	0.031
ADG, g	346^a,b^	273^b^	361^a,b^	398^a^	15.687	0.026
ADFI, g	620	557	646	660	23.259	0.429
G:F	0.28^a,b^	0.25^b^	0.28^a,b^	0.30^a^	0.006	0.043
Diarrhea score	0.79^a,b^	0.99^a^	0.58^b^	0.58^b^	0.059	0.024

*BW* Body weight, *ADG* Average daily gain, *ADFI* Average daily feed intake, *G:F* Ggain-to-feed ratio

^1^Data represent the mean of 6 replicate pens per treatment

^2^CON: basal diet + saline solution; NC: basal diet + ETEC K88 challenge; PC: basal diet + 2,500 mg/kg of Zn (ZnO) + ETEC K88 challenge; ZnCA: basal diet + 1,600 mg/kg of Zn (ZnCA) + ETEC K88 challenge

^3^SEM: standard error of the mean

^a,b^Means with similar superscripts within the same row showed no significant difference (*P* > 0.05)

Both the ZnCA (*P* = 0.042) and PC (*P* = 0.040) groups had significantly lower fecal scores than the NC group. However, no significant difference was noted between piglets in the CON group and those in the other treatment groups.

The impact of dietary ZnCA supplementation on the organ indexes of piglets is presented in Additional file 2. The weight of the kidneys, spleen, heart, and liver remained unaffected by the dietary treatments (*P* > 0.05).

### Intestinal health (intestinal histomorphometry, ATTD of nutrients, and gut barrier)

**Fig. 1:**
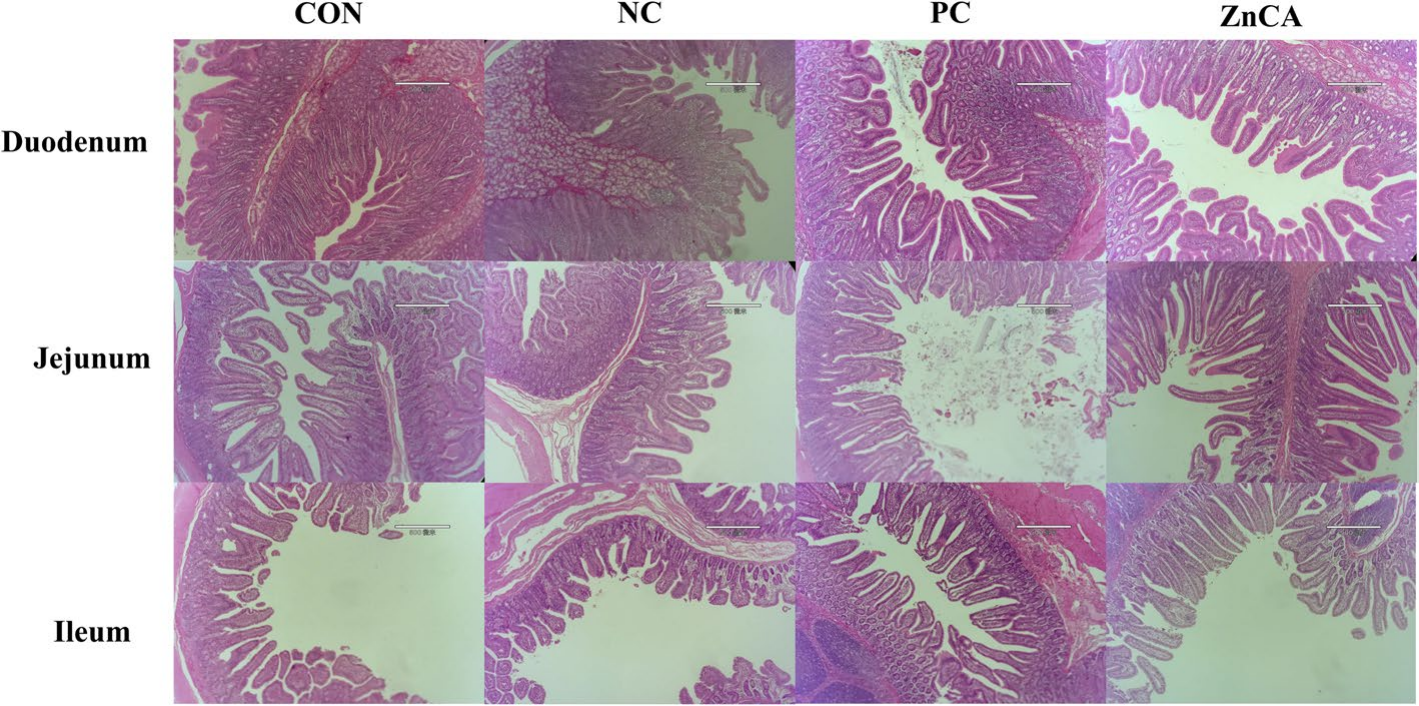
Effects of dietary ZnCA supplementation on intestinal morphology in piglets. All intestinal tissue samples were examined using H&E staining, and images were captured at a magnification of 40 × using an optical microscope. CON: basal diet + saline solution; NC: basal diet + ETEC K88 challenge; PC: basal diet + 2,500 mg/kg of Zn (ZnO) + ETEC K88 challenge; ZnCA: basal diet + 1,600 mg/kg of Zn (ZnCA) + ETEC K88 challenge

As depicted in Fig. 1 and Table 3, there was no statistically significant variance in duodenum morphology across the four groups (*P* > 0.05). In both the ileum and jejunum, the ZnCA group demonstrated a higher villus height and villus/crypt ratio as well as a lower crypt depth than the CON and NC groups (*P* < 0.05). The PC group demonstrated an increased villus/crypt ratio in the jejunum and villus height in the ileum along with a decreased crypt depth in the jejunum when compared with the CON and NC groups (*P* < 0.05). Notably, the NC group exhibited a significantly lower jejunal villus/crypt ratio (*P* = 0.033) and higher jejunal villus height (*P* = 0.020) than the CON group.

**Table 3:** Effects of dietary ZnCA supplementation on intestinal histomorphometry in piglets^1^

Parameters	Dietary treatments^2^				SEM^3^	*P*-value
	CON	NC	PC	ZnCA		
Duodenum						
Villus height, µm	318.46	296.76	315.67	309.89	4.442	0.428
Crypt depth, µm	184.89	179.11	178.93	176.72	3.322	0.872
Villus/crypt ratio	1.72	1.68	1.79	1.79	0.024	0.316
Jejunum						
Villus height, µm	301.57^b^	284.73^b^	307.03^b^	371.83^a^	4.678	< 0.001
Crypt depth, µm	128.77^a^	131.15^a^	101.19^b^	109.69^b^	2.069	< 0.001
Villus/crypt ratio	1.56^c^	1.42^d^	2.05^b^	2.48^a^	0.045	< 0.001
Ileum						
Villus height, µm	250.12^c^	230.74^d^	270.65^b^	289.66^a^	2.876	< 0.001
Crypt depth, µm	109.34^a,b^	100.05^b^	110.79^a,b^	118.85^a^	1.808	0.003
Villus/crypt ratio	2.33^b^	2.34^b^	2.46^a,b^	2.51^a^	0.020	0.001

^1^Data represent the mean of 6 replicate pens per treatment

^2^CON: basal diet + saline solution; NC: basal diet + ETEC K88 challenge; PC: basal diet + 2,500 mg/kg of Zn (ZnO) + ETEC K88 challenge; ZnCA: basal diet + 1,600 mg/kg of Zn (ZnCA) + ETEC K88 challenge

^3^SEM: standard error of the mean

^a−d^Means with similar superscripts within the same row showed no significant difference (*P* > 0.05)

The ATTD of nutrients in piglets is summarized in Table 4. There were no statistically significant differences detected between the PC and ZnCA groups (*P* > 0.05). The addition of ZnO and ZnCA increased the digestibility of DM and GE (*P* < 0.05). Moreover, the digestibility of crude protein in the NC group was notably lower than that in the CON group (*P* = 0.001).

**Table 4:** Effects of dietary ZnCA supplementation on the ATTD of nutrients in piglets^1^

ATTD, %	Dietary treatments^2^				SEM^3^	*P*-value
	CON	NC	PC	ZnCA		
Dry matter	75.357^b^	75.273^b^	79.6583^a^	79.710^a^	0.763	0.029
Crude protein	68.837^a^	67.083^b^	69.817^a^	69.818^a^	0.267	< 0.001
Gross energy	71.718^b^	66.866^b^	77.332^a^	77.3214^a^	1.088	< 0.001

*ATTD* Apparent total tract digestibility

^1^Data represent the mean of 6 replicate pens per treatment

^2^CON: basal diet + saline solution; NC: basal diet + ETEC K88 challenge; PC: basal diet + 2,500 mg/kg of Zn (ZnO) + ETEC K88 challenge; ZnCA: basal diet + 1,600 mg/kg of Zn (ZnCA) + ETEC K88 challenge

^3^SEM: standard error of the mean

^a,b^Means with similar superscripts within the same row showed no significant difference (*P* > 0.05)

The serum concentrations of D-LA, DAO, and endotoxin—which are indicators of intestinal permeability—were assayed (Table 5). No significant differences in the serum levels of DAO were observed among the treatment groups (*P* > 0.05). However, the levels of D-LA and endotoxin were significantly higher in the CON group than in the other experimental groups (*P* < 0.05). Interestingly, there was no notable distinction in serum D-LA and endotoxin levels between the CON and ZnCA groups (*P* > 0.05). Meanwhile, in comparison to the PC group, the ZnCA group demonstrated reduced serum levels of D-LA and endotoxin (*P* < 0.05).

**Table 5:** Effects of dietary ZnCA supplementation on intestinal barrier function in piglets^1^

Parameters	Dietary treatments^2^				SEM^3^	*P*-value
	CON	NC	PC	ZnCA		
Serum indicators of intestinal barrier function						
D-LA, μmol/mL	3.51^b,c^	4.86^a^	4.02^b^	3.22^c^	0.159	< 0.001
DAO, U/L	152.549	129.79	146.67	150.16	5.908	0.547
Endotoxin, ng/mL	316.14^c^	535.15^a^	439.38^b^	313.79^c^	20.616	< 0.001
Expression of intestinal barrier-related genes in the jejunum mucosa						
*ZO-1*	1.058^a^	0.76^b^	0.930^ab^	1.116^a^	0.039	0.007
*MUC-2*	1.029^b,c^	1.237^a,b^	0.884^c^	1.363^a^	0.043	< 0.001
Occludin	1.018^b^	1.141^b^	1.093^b^	1.516^a^	0.047	< 0.001
Claudin-1	1.122^b^	0.225^c^	1.203^b^	2.811^a^	0.104	< 0.001
Claudin-2	1.021^b^	1.449^a^	1.086^b^	1.125^b^	0.028	< 0.001

*D-LA* D-lactic acid, *DAO* Diamine oxidase, *ZO-1* Zonula occludens-1, *MUC-2* Mucin 2

^1^Data represent the mean of 6 replicate pens per treatment

^2^CON: basal diet + saline solution; NC: basal diet + ETEC K88 challenge; PC: basal diet + 2,500 mg/kg of Zn (ZnO) + ETEC K88 challenge; ZnCA: basal diet + 1,600 mg/kg of Zn (ZnCA) + ETEC K88 challenge

^3^SEM: standard error of the mean

^a−c^Means with similar superscripts within the same row showed no significant difference (*P* > 0.05)

The relative mRNA levels of tight junction (TJ) proteins within the jejunum were also examined (Table 5). Notably, ZnCA supplementation significantly upregulated the mRNA levels of mucin 2 (*MUC-2*), Occludin, and Claudin-1 in the jejunum. Interestingly, no notable differences in the mRNA levels of TJ proteins were detected between the CON and PC groups (*P* > 0.05). However, the NC group exhibited lower mRNA levels of Claudin-1 and *ZO-1*, as well as higher mRNA level of Claudin-2 (*P* > 0.05).

### Anti-inflammatory activity

The anti-inflammatory activity of dietary ZnCA supplementation is detailed in Table 6. Initially, we assessed serum liver function markers, including AKP, AST/GOT, and ALT/GPT. However, no notable variances were detected in the serum levels of AST/GOT and ALT/GPT across all groups (*P* > 0.05).

**Table 6:** Effects of dietary ZnCA supplementation on anti-inflammatory activity in piglets^1^

Parameters	Dietary treatments^2^				SEM^3^	*P*-value
	CON	NC	PC	ZnCA		
Serum liver function						
AKP, U/L	33.76^a,b^	19.29^c^	43.44^a^	32.52^b^	1.762	< 0.001
AST/GOT, U/L	15.91	10.60	14.40	12.80	1.017	0.289
ALT/GPT, U/L	25.84	27.10	24.09	29.36	1.101	0.408
Serum cytokines						
IL-6, pg/mL	111.93^b^	158.29^a^	122.79^b^	108.47^b^	4.866	< 0.001
IL-1β, pg/mL	301.68^c^	437.28^a^	387.02^a,b^	312.13^b,c^	14.741	< 0.001
TNF-α, pg/mL	58.68^c^	109.99^a^	66.64^b,c^	83.55^b^	4.942	< 0.001
NO, μmol/L	106.26^a,b^	130.12^a^	77.51^b^	77.11^b^	7.018	0.007
Inflammatory factors in the jejunum mucosa						
*IL-6*	1.123^b,c^	2.376^a^	1.170^b^	0.5719^c^	0.109	< 0.001
*IL-1β*	1.018^b^	2.391^a^	0.956^b^	1.018^b^	0.092	< 0.001
*TNF-α*	1.053^b^	4.008^a^	1.461^b^	1.740^b^	0.222	< 0.001
*iNOS*	1.087^c^	3.328^a^	2.092^b^	1.250^c^	0.140	< 0.001
Inflammatory factors in the liver						
*IL-6*	1.068^b^	2.280^a^	1.106^b^	0.923^b^	0.112	< 0.001
*IL-1β*	1.084^b^	3.599^a^	1.652^b^	1.116^b^	0.172	< 0.001
*TNF-α*	1.032^b^	3.133^a^	1.092^b^	1.136^b^	0.155	< 0.001
*iNOS*	1.161^b^	3.413^a^	1.844^b^	1.083^b^	0.158	< 0.001

*IL-6* Interleukin-6, *IL-1β* Interleukin-1β, *TNF-α* Tumor necrosis factor-α, *NO* Nitric oxide, *AKP* Alkaline phosphatase, *AST/GOT* Aspartate aminotransferase, *ALT/GPT* Alanine aminotransferase, *iNOS* Inducible nitric oxide synthase

^1^Data represent the mean of 6 replicate pens per treatment

^2^CON: basal diet + saline solution; NC: basal diet + ETEC K88 challenge; PC: basal diet + 2,500 mg/kg of Zn (ZnO) + ETEC K88 challenge; ZnCA: basal diet + 1,600 mg/kg of Zn (ZnCA) + ETEC K88 challenge

^3^SEM: standard error of the mean

^a−c^Means with similar superscripts within the same row showed no significant difference (*P* > 0.05)

Interestingly, the serum levels of IL-6, NO, IL-1β, and TNF-α were notably decreased after the NC treatment (*P* < 0.05). However, notable differences in the serum levels of the cytokines IL-6, NO, and IL-1β were observed between the CON and ZnCA groups (*P* > 0.05). When compared to CON treatment, PC treatment produced higher serum concentrations of IL-1β (*P* < 0.05) but did not alter those of IL-6, TNF-α, and NO (*P* > 0.05).

Consistent with the serum cytokine levels, the mRNA levels of inflammatory cytokines—such as *IL-6*, *iNOS*, *TNF-α*, and *IL-1β*—were markedly elevated in the jejunum mucosa and liver following NC treatment (*P* < 0.05). However, no notable differences in inflammatory factor mRNA levels in both the jejunum mucosa and the liver were observed between the CON and ZnCA groups (*P* > 0.05). Interestingly, the PC group demonstrated elevated mRNA levels of *IL-6* and *iNOS* when compared with the ZnCA group (*P* < 0.05).

### Zn homeostasis

The impact of dietary ZnCA supplementation on the homeostasis of trace metals in piglets is shown in Table 7. Notably, a positive correlation was observed between the Zn concentration in the feces, liver, and kidney and the Zn concentration in the feed (*P* < 0.05). Furthermore, serum zinc concentrations were markedly higher in the PC group than in both the CON and NC groups (*P* < 0.05). However, no notable differences in serum Zn concentrations and the Zn concentrations in the musculus longissimus dorsi and hair were observed between the ZnCA group and the other three treatment groups (*P* > 0.05). Notably, the Zn concentration in the jejunum was lower in the NC group than in the other treatment groups (*P* > 0.05).

**Table 7:** Effects of dietary ZnCA supplementation on the homeostasis of trace metals in piglets^1^

Parameters^4^	Dietary treatments^2^				SEM^3^	*P*-value
	CON	NC	PC	ZnCA		
Zn concentration						
Feed, mg/kg	128.93^c^	128.93^c^	2508.30^a^	1607.71^b^	306.27	< 0.001
Feces, g/kg	1.644^c^	1.548^c^	24.815^a^	15.748^b^	2.251	< 0.001
Serum, mg/L	2.842^b^	3.208^b^	5.558^a^	4.781^a,b^	0.332	0.003
Liver, mg/kg	201.91^c^	197.78^c^	1346.57^a^	907.44^b^	108.26	< 0.001
Kidney, mg/kg	109.36^c^	114.62^c^	396.31^a^	202.94^b^	29.088	< 0.001
Jejunum, mg/kg	196.66^a^	130.00^b^	199.64^a^	215.89^a^	8.604	< 0.001
Musculus longissimus dorsi, mg/kg	73.608	77.103	73.627	74.052	1.683	0.878
Hair, mg/kg	1192.88	1086.23	1092.47	1090.28	23.001	0.298
Cu concentration						
Feed, mg/kg	24.053	24.053	23.750	24.337	0.602	0.759
Feces, mg/kg	272.37^b^	246.84^b^	377.08^a^	285.32^b^	11.334	< 0.001
Serum, mg/L	3.161	4.054	3.257	3.552	0.138	0.085
Liver, mg/kg	24.700^a,b^	31.117^a^	19.183^b^	24.754^a,b^	1.165	0.001
Kidney, mg/kg	40.650^b,c^	32.792^c^	121.895^a^	75.102^b^	8.577	< 0.001
Jejunum, mg/kg	18.192^a^	13.658^b,d^	17.029^a,b^	11.613^c,d^	0.758	0.002
Musculus longissimus dorsi, mg/kg	7.879^a^	7.250^a,b^	5.329^b^	5.613^a,b^	0.351	0.014
Hair, mg/kg	8.618^a^	7.521^b^	7.137^b^	7.691^a,b^	0.164	0.004
Fe concentration						
DM of feed, mg/kg	309.67	309.67	323.29	322.94	2.927	0.137
DM of feces, mg/kg	2,933.61^b^	2,954.32^b^	4,163.78^a^	3,405.58^a,b^	153.12	0.005
Serum, mg/L	12.283	12.987	11.904	10.627	0.351	0.106
Liver, mg/kg	135.59	130.53	148.78	144.33	7.524	0.884
Kidney, mg/kg	136.11	131.19	135.38	134.88	4.182	0.986
Jejunum, mg/kg	228.90^a^	198.54^a,b^	181.01^b^	174.77^b^	7.055	0.019
Musculus longissimus dorsi, mg/kg	157.38^a^	125.35^a,b^	110.55^b^	93.56^b^	6.294	< 0.001
Hair, mg/kg	615.78	471.93	648.56	523.88	41.08	0.421
Mn concentration						
Feed, mg/kg	43.034	43.034	47.247	45.890	0.798	0.139
Feces, mg/kg	443.87	442.88	536.42	470.94	16.183	0.132
Liver, mg/kg	6.313	5.746	4.501	5.375	0.332	0.284
Kidney, mg/kg	6.542	4.985	5.330	4.815	0.264	0.077
Hair, mg/kg	18.275	17.275	17.333	16.521	0.862	0.925

^1^Data represent the mean of 6 replicate pens per treatment

^2^CON: basal diet + saline solution; NC: basal diet + ETEC K88 challenge; PC: basal diet + 2,500 mg/kg of Zn (ZnO) + ETEC K88 challenge; ZnCA: basal diet + 1,600 mg/kg of Zn (ZnCA) + ETEC K88 challenge

^3^SEM: standard error of the mean

^4^With the exception of the serum, the levels in all other samples were calculated based on dry matter

^a−d^Means with similar superscripts within the same row showed no significant difference (*P* > 0.05)

There were no notable variances in the Cu and Fe concentrations of the feed among the groups (*P* > 0.05). Interestingly, the PC group exhibited higher levels of Cu and Fe in the feces than the other treatment groups (*P* < 0.05). A trend towards increased serum Cu concentrations was also detected in the NC treatment group (*P* = 0.085). In comparison to the CON group, the PC group exhibited elevated levels of Cu in the kidneys and reduced levels of Cu in the musculus longissimus dorsi and hair (*P* < 0.05). Additionally, the NC group demonstrated higher hepatic levels of Cu (*P* < 0.05). There were no notable differences in the Cu concentrations in the liver, kidney, musculus longissimus dorsi, and hair between the ZnCA and CON treatment groups (*P* > 0.05). Notably, the ZnCA treatment group had lower Cu levels in the jejunum than the CON and PC treatment groups (*P* < 0.05).

Furthermore, no notable variances were detected in the Fe concentrations of the liver, kidneys, and hair (*P* > 0.05). The CON group exhibited higher Fe levels in the jejunum and musculus longissimus dorsi than the PC and ZnCA groups (*P* < 0.05). Although the Mn concentration of the feed, feces, liver, and hair was comparable across the various treatment groups (*P* > 0.05), the Mn concentration in the kidneys tended to show variations (*P* = 0.077).

The relative mRNA levels of Zn/iron-regulated transporter-like 5 (*ZIP5*) remained unaffected (*P* > 0.161) in the kidneys, liver, and jejunum mucosa, as presented in Table 8. Meanwhile, the mRNA expression of *ZIP4* in the jejunum mucosa was altered in both the NC and PC groups (*P* < 0.001). Notably, the NC group exhibited the highest relative mRNA levels of *ZIP14* in the jejunum mucosa and liver (*P* < 0.001). With regard to the mRNA expression of Zn transporter 1 (*ZNT1*) in the jejunum mucosa, liver, and kidney, no significant variances were detected between the CON and NC treatment groups (*P* > 0.05). However, the PC group demonstrated the highest mRNA levels of *ZNT1* across all these three tissues (*P* < 0.05). The ZnCA group exhibited similar mRNA levels of *ZNT1* in the jejunum mucosa as the PC group, but the hepatic and renal expression levels of this gene were lower in the ZnCA group (*P* < 0.05). Furthermore, significant variances in the mRNA expression of metallothionein 1 (*MT1*), *MT2*, and *MT3* were observed among the four dietary treatments (*P* < 0.05). Specifically, the PC treatment group showed the highest levels of these genes, with the ZnCA group following closely behind. In contrast, the expression of these genes in the kidneys, liver, and jejunum mucosa was lower in the CON and NC treatment groups (*P* < 0.05). Finally, no notable variations were observed in the mRNA expression of *ZIP8* in the liver tissue among the four treatment groups (*P* > 0.05).

**Table 8:** Effects of dietary ZnCA supplementation on the relative mRNA levels of specific Zn transporters in piglets^1^

Genes	Dietary treatments^2^				SEM^3^	*P*-value
	CON	NC	PC	ZnCA		
Jejunum mucosa						
*ZIP4*	1.039^b^	2.626^a^	0.197^c^	0.609^b,c^	0.142	< 0.001
*ZIP5*	1.060	1.105	1.064	1.065	0.043	0.984
*ZIP14*	1.145^b^	2.628^a^	1.734^b^	0.904^b^	0.142	< 0.001
*ZNT1*	1.048^b^	0.892^b^	1.273^a^	1.401^a^	0.044	< 0.001
*MT1*	1.042^c^	0.513^c^	158.632^a^	57.965^b^	9.174	< 0.001
*MT2*	1.037^c^	0.957^c^	102.363^a^	53.122^b^	3.962	< 0.001
*MT3*	1.359^b^	1.196^b^	747.351^a^	103.034^b^	35.545	< 0.001
Liver						
*ZIP5*	0.989	1.046	1.059	1.052	0.392	0.929
*ZIP8*	1.204	1.413	1.376	1.001	0.074	0.160
*ZIP14*	1.009^b^	2.233^a^	1.150^b^	1.018^b^	0.095	< 0.001
*ZNT1*	1.193^c^	1.274^c^	3.251^a^	2.198^b^	0.117	< 0.001
*MT1*	1.055^c^	0.618^c^	35.232^a^	14.245^b^	1.505	< 0.001
*MT2*	1.050^c^	1.531^c^	21.362^a^	8.880^b^	1.044	< 0.001
*MT3*	1.284^c^	0.935^c^	284.354^a^	66.097^b^	17.453	< 0.001
Kidney						
*ZIP5*	1.057	1.297	1.030	1.050	0.041	0.161
*ZNT1*	1.071^b^	1.212^b^	2.011^a^	1.062^b^	0.085	< 0.001
*MT1*	1.265^c^	1.754^c^	34.989^a^	8.809^b^	1.535	< 0.001
*MT2*	1.142^c^	1.518^c^	22.805^a^	7.588^b^	1.089	< 0.001
*MT3*	1.033^c^	1.132^c^	7.297^a^	4.652^b^	0.372	< 0.001

*ZIP4* Zn/iron-regulated transporter-like 4, *ZIP5* Zn/iron-regulated transporter-like 5, *ZIP8* Zn/iron-regulated transporter-like 8, *ZIP14* Zn/iron-regulated transporter-like 14, *MT1* Metallothionein 1, *MT2* Metallothionein 2, *MT3* Metallothionein 3, *ZNT1* Zn transporter 1

^1^Data represent the mean of 6 replicate pens per treatment

^2^CON: basal diet + saline solution; NC: basal diet + ETEC K88 challenge; PC: basal diet + 2,500 mg/kg of Zn (ZnO) + ETEC K88 challenge; ZnCA: basal diet + 1,600 mg/kg of Zn (ZnCA) + ETEC K88 challenge

^3^SEM: standard error of the mean

^a−c^Means with similar superscripts within the same row showed no significant difference (*P* > 0.05)

## Discussion

Due to the combined stress of weaning, incomplete intestinal development, and the influence of the rearing environment, piglets frequently develop PWD. This condition can lead to diarrhea, decreased feed intake, growth retardation, and even mortality among weaned piglets [28]. In context of the dual challenges arising from the ban on AGPs and the limitations imposed upon Zn supplementation, organic Zn has emerged as a promising solution for PWD management [29].

The impact of pharmacological doses of ZnO on nutrient digestibility, diarrhea incidence, and growth performance in weaned piglets has been studied extensively [30–32]. In the current study, dietary ZnO supplementation (2,500 mg/kg) significantly reduced the diarrhea score in weaned piglets challenged with ETEC K88, but it did not improve their growth performance. However, these findings contrast with those reported by Lei and Kim [17], who observed that 2,500 mg/kg of Zn in the form of ZnO enhance growth performance in weaned piglets exposed to ETEC K88. This discrepancy could arise because Lei and Kim administered a high-Zn diet to the piglets for 21 d prior to the ETEC K88 challenge.

Our previous study demonstrated that zinc laurate (ZnLa) can effectively alleviate intestinal symptoms in mice exposed to ETEC K88 [33]. Additionally, we noted that ZnCA exhibits significantly greater antibacterial activity than ZnLa [23]. Consequently, we hypothesized that ZnCA may effectively alleviate intestinal dysfunction and enhance growth performance in weaned piglets challenged with ETEC K88. As expected, in the present study, piglets who received a diet containing an additional 1,600 mg/kg of Zn in the form of ZnCA exhibited a higher ADG and G:F ratio as well as a lower fecal score after the ETEC K88 challenge than the piglets who were fed a basal diet. These results confirmed that compared with the addition of pharmacological doses of ZnO, incorporating a relatively lower level of ZnCA into the diet can promote growth and reduce diarrhea in weaned piglets, offering results comparable to those achieved with pharmacological doses of ZnO.

After weaning and exposure to ETEC K88, piglets often experience alterations in the structure and functionality of the intestinal tract. These changes primarily manifest in the form of villus atrophy and crypt hyperplasia [34]. Previous research has established that pharmacological ZnO supplements can improve the intestinal structure in weaned piglets [16, 35, 36]. In accordance with these results, our findings demonstrated that both 2,500 mg/kg of conventional ZnO and 1,600 mg/kg of ZnCA can improve the ratio of villus height to crypt depth and reduce crypt depth in the jejunum. Moreover, ZnCA supplementation was observed to enhance villus height and the villus/crypt ratio in the ileum, while also decreasing crypt depth. Meanwhile, ZnO supplementation also increased the villus/crypt ratio in the ileum. Notably, 1,600 mg/kg ZnCA improved the villus/crypt ratio in the jejunum to a greater degree than the pharmacological doses of ZnO, indicating a potential enhancement of absorption capacity in the intestine following ZnCA supplementation.

The villi of the small intestine play a key role in nutrient absorption. Hence, PWD typically reduces the nutrient absorption capacity in affected animals [34]. In this study, piglets challenged with ETEC K88 and treated with ZnCA exhibited an improvement in intestinal structure, resulting in the increased ATTD of DM, CP, and GE. Similar findings have also been reported by Lei and Kim [17]. This increase in the digestibility of DM, CP, and GE observed in ZnCA-treated piglets suggests that improved nutrient digestibility is at least partly responsible for enhanced growth performance in these animals [17]. This potential increase in the rate of nutrient digestion can be attributed to improvements in intestinal tract structure [37]. Furthermore, previous studies conducted by Hedemann et al. [38] and Hu et al. [39] indicate that pharmacological doses of ZnO can enhance the activity of digestive enzymes in the intestines of weaned piglets. Therefore, increased enzyme activity may also contribute to the accelerated rate of nutrient digestion observed in these piglets.

The onset of PWD is intricately linked to intestinal permeability [40, 41]. TJ proteins—such as Occludin, Claudin-1, and ZO-1—play an important role in regulating the permeability of intestinal epithelial cells and maintaining barrier function [42]. In this study, we observed that infection with ETEC significantly reduced the mRNA levels of Claudin-1 and *ZO-1* in the jejunal mucosa of weaned piglets. However, supplementation with ZnCA could alleviate this damage and partially increase the mRNA expression of TJ proteins. Consistent with our findings, Xie et al. [15] discovered that the addition of polysaccharide-Zn complexes to the diet can upregulate the mRNA expression of TJ proteins.

Claudin-2, a pore-forming protein, is known to disrupt the TJ barrier [43]. Notably, in our study, the mRNA levels of Claudin-2 exhibited an inverse trend compared to those of Claudin-1, echoing the similar antagonistic effects reported by Jung et al. [44].

As reliable indicators of intestinal permeability, the levels of D-LA, DAO, and endotoxin provide key insights into intestinal barrier function [45]. An investigation conducted by Xu et al. [46] revealed that supplementation with 1,600 mg/kg of ZnO can effectively reduce DAO and endotoxin levels in the serum. In our study, we observed that ETEC K88 could significantly upregulate the serum concentrations of D-LA and endotoxin. However, pharmacological doses of ZnO and 1,600 mg/kg of ZnCA could mitigate these elevations, with ZnCA demonstrating superior efficacy. These findings suggest that ZnCA, which also reduces the required amount of Zn supplementation, enhances intestinal health in weaned piglets challenged with ETEC K88 through various mechanisms. These mechanisms include improvements in intestinal morphology, augmented nutrient digestibility, and reinforcement of intestinal barrier function. Notably, the efficacy of ZnCA surpasses that of pharmacological ZnO doses in achieving these benefits.

After weaning, piglets frequently experience inflammation due to dietary transitions and infections caused by pathogens such as ETEC [47]. AKP plays a crucial role in detoxification and anti-inflammatory processes following ETEC infections [48]. In this study, exposure to ETEC K88 in weaned piglets led to decreased levels of AST/GOT and ALT/GPT and significantly suppressed AKP activity in the serum. Conversely, Zn supplementation could enhance AKP activity, likely because AKP is a Zn-containing metalloenzyme and Zn augments its functionality [49]. Comparable findings have been documented by Liu et al. [50]. Our previous research demonstrated the anti-inflammatory effects of Zn laurate in mice infected with ETEC K88 [33]. Therefore, in the present study, we further explored the impact of ZnCA on inflammatory markers in post-weaned piglets challenged with ETEC K88. Akin to pharmacological doses of ZnO, ZnCA notably decreased the mRNA levels of *iNOS*, *IL-6*, *TNF-α*, and *IL-1β* in the liver and jejunum mucosa, as well as the levels of NO, IL-6, TNF-α, and IL-1β in the serum of weaned piglets challenged with ETEC K88. Evidence suggests that MCFAs possess immunomodulatory properties and may improve the overall health of weaned piglets [51]. Consequently, the anti-inflammatory effects of ZnCA on weaned piglets could be attributed to the synergistic actions of MCFAs and Zn.

So far, studies on organic and inorganic Zn in weaned piglets have explored the mechanisms of Zn metabolism and the anti-inflammatory effects of Zn independent of each other. Previous studies suggest that Zn can modulate the activity of inflammatory pathways [52]. These inflammatory processes can, in turn, influence the expression of Zn transporters [53]. Hence, one key objective of the present study was to examine the relationship between the anti-inflammatory activity of ZnCA and Zn metabolism following ZnCA administration, offering insights into their interrelationships.

ZIP4, which is a Zn transporter, is predominantly expressed in the brush border of the intestines and facilitates the absorption of Zn from the intestinal lumen [54]. In this study, the mRNA levels of *ZIP4* were found to be the highest in the NC treatment group and the lowest in the PC treatment group. The downregulation of *ZIP4* in the intestinal mucosa of piglets in the PC and ZnCA groups can be attributed to the mechanism of Zn absorption at saturation [55], wherein the rate of intestinal absorption is inversely correlated with the intake [56]. Conversely, the overexpression of *ZIP4* mRNA in the NC group may be due to the pathogenic effects of ETEC K88 on the intestines, resulting in decreased Zn absorption and a compensatory upregulation of *ZIP4*. Nevertheless, alterations in absorption rates do not necessarily reflect changes in total nutrient absorption [57]. Despite the decreased absorption rate, the Zn content in the jejunum of the PC and ZnCA treatment groups remained significantly higher than that in the jejunum of the CON group. Conversely, the jejunum Zn content in the NC group continued to remain the lowest among all the groups, consistent with previous findings [58].

*ZIP5*, which is integral for Zn uptake from the bloodstream, did not exhibit altered expression levels in the small intestinal mucosa, liver, and kidneys in the present study. This contradicts the results reported by Dalto et al. [59], and these differences are likely due to variations in the feeding duration between the two studies.

*ZIP8* transcription is directly modulated by the NF-κB signaling pathway [53]. However, our findings revealed no notable changes in *ZIP8* mRNA expression in the liver under the different treatment conditions, possibly due to the tissue-specific nature of this mRNA [60]. Unlike ZIP8, ZIP14 can suppress NF-κB signaling via a negative feedback loop [61]. In the present study, *ZIP14* mRNA levels showed a significant increase in the NC group, in contrast to the downregulation of *ZIP14* in the PC and ZnCA groups. This suggests that *ZIP14* expression may primarily be governed by inflammatory factors and not Zn regulation [62].

ZNT1 is a basolateral membrane transporter for Zn in enterocytes. The mRNA expression of *ZNT1* is positively correlated with tissue Zn levels. Following excessive Zn intake, *ZNT1* expression increases to facilitate Zn excretion. Therefore, it is evident that *ZNT1* expression is primarily regulated by Zn levels. Additionally, Nishito and Kambe [63] delineated the cooperative roles of ZNT1 and MT in maintaining cellular Zn homeostasis. In the present study, we observed similar trends in *MT1*, *MT2*, and *MT3* expression, consistent with the changes in *ZNT1* expression. Wang et al. [64] reported that the kidneys play a crucial role in Zn excretion when dietary Zn intake becomes high. However, in the present study, the mRNA levels of *ZNT1* in the kidneys of weaned piglets subjected to ZnCA treatment were not significantly different from those in the CON group. Consequently, unlike the administration of 2,500 mg/kg of ZnO, supplementation with 1,600 mg/kg of ZnCA appeared to have minimal impact on Zn homeostasis in the kidneys.

The body maintains Zn homeostasis through both a rapid exchange pool (encompassing the serum, intestines, liver, and kidneys) and a slow exchange pool (including skeletal muscles, bones, and hair) [65]. Our findings revealed that ZnO and ZnCA primarily influence the Zn content within the tissues encompassing the rapid exchange pool. Although the ZnCA group received a lower dose of zinc supplementation than the PC group, jejunal zinc levels were comparable between the ZnCA and PC groups. Moreover, the PC group demonstrated higher hepatic and renal zinc accumulation, as well as greater levels of zinc excretion, than the ZnCA group. Oh et al. [29] suggested that organic zinc has a higher absorption rate in the body than inorganic zinc. However, given that the evidence from the present study is insufficient to appropriately reach this conclusion, our future studies will seek to explore whether ZnCA exhibits a higher absorption rate than inorganic zinc. Nevertheless, the regulation of Zn homeostasis can affect the metabolism of other minerals, such as Cu and Fe. Matte et al. [66] observed that an increase in dietary Zn intake can reduce Cu efflux from intestinal cells. Consistent with these findings, in the PC group, the concentration of Cu was elevated in the jejunum but reduced in the liver and serum. Additionally, our study revealed that high-Zn diets can enhance the excretion of Cu and Fe. This conclusion was corroborated by the findings reported by Foligné et al. [67]. Elevations in Zn content likely contribute to this phenomenon by upregulating the expression of MT [68], which can bind to Fe and Cu and thereby limit their transport into the blood and liver [69]. Ultimately, the Cu and Fe bound to MT can be eliminated via the shedding of intestinal cells [59]. Notably, supplementation with 1,600 mg/kg ZnCA only decreased duodenal Cu and musculus longissimus dorsi Fe levels in weaned piglets, indicating that ZnCA had a significantly lower metabolic impact on Cu and Fe than pharmacological levels of ZnO. Finally, neither dietary manipulation nor toxin exposure was found to produce any discernible effect on Mn metabolism in this study, suggesting that high Zn supplementation may not alter Mn metabolism.

## Conclusions

The findings of the current study demonstrate that incorporating 1,600 mg/kg of ZnCA into the diet of weaned piglets can lead to improved growth performance and nutrient digestibility, while also reducing the incidence of diarrhea and inflammatory responses triggered by ETEC K88 infection. These beneficial effects are comparable to those achieved with pharmacological doses of ZnO. Furthermore, in terms of improving intestinal health and Zn homeostasis, the efficacy of 1,600 mg/kg ZnCA surpasses that of pharmacological doses of ZnO in weaned piglets challenged with ETEC K88.

Pharmacological doses of ZnO not only increase Zn excretion but also disrupt the absorption of copper and iron. However, our study revealed that the ZnCA (1,600 mg/kg) can achieve reduced Zn substitution while still enhancing Zn deposition in weaned piglets. Our future studies will involve a deeper exploration of the impact of lower ZnCA doses on weaned piglets. Given the current restrictions regarding the pharmacological dosage of ZnO, this study offers valuable insights for reducing Zn excretion and presents a novel approach towards managing PWD and developing antibiotic alternatives for weaned piglets.

## Supplementary Information

Additional file 1. Primer sequences used for RT-qPCR amplification.

Additional file 2. Effects of dietary ZnCA supplementation on organ indexes in piglets.

## Data Availability

The datasets used in the current study are available from the corresponding author on reasonable request.

## Abbreviations

ADFI: Average daily feed intake
ADG: Average daily gain
AKP: Alkaline phosphatase
ALT/GPT: Glutamic-pyruvic transaminase
AST/GOT: Glutamic-oxaloacetic transaminase
ATTD: Apparent total tract digestibility
CA: Caproic acid
CP: Crude protein
DAO: Diamine oxidase
D-LA: D-Lactic acid
DM: Dry matter
ETEC: *Escherichia coli*
GAPDH: Glyceraldehyde 3-phosphate dehydrogenase
GE: Gross energy
G:F: Feed efficiency
IL-1β: Interleukin-1β
IL-6: Interleukin-6
iNOS: Inducible nitric oxide synthase
MCFAs: Medium-chain fatty acids
MT1: Metallothionein 1
MT2: Metallothionein 2
MT3: Metallothionein 3
MUC-2: Mucin 2
NO: Nitric oxide
PWD: Post-weaning diarrhea
TNF-α: Tumor necrosis factor-α
ZIP4: Zn/iron-regulated transporter-like 4
ZIP5: Zn/iron-regulated transporter-like 5
ZIP8: Zn/iron-regulated transporter-like 8
ZIP14: Zn/iron-regulated transporter-like 14
ZnCA: Zinc caproate
ZnO: Zinc oxide
ZNT1: Zn transporter 1
ZO-1: Zonula occludens-1
