# Supplementary material for: Impacts of zinc caproate supplementation on growth performance, intestinal health, anti-inflammatory activity, and Zn homeostasis in weaned piglets challenged with Escherichia coli K88
Source: J Anim Sci Biotechnol. 2025 Mar 14;16:44. doi: 10.1186/s40104-025-01172-2 (PMC11908000; doi:10.1186/s40104-025-01172-2)
Supplement: Supplementary file 1 — Additional file 1. Primer sequences used for RT-qPCR amplification. [file 40104_2025_1172_MOESM1_ESM.doc]

Additional file 1. Primer sequences used for RT-qPCR amplification

Table S1. Primer sequences used for RT-qPCR amplification

| Gene symbol^1^ | Primer sequences (5´– 3´) | GenBank accession no. |
| --- | --- | --- |
| Housekeeping genes | | |
| *GAPDH* | CCAAGGAGTAAGAGCCCCTG | XM_021091114.1 |
|  | AAGTCAGGAGATGCTCGGTG |  |
| Genes related to intestinal barrier integrity | | |
| *ZO-1* | CAGCACAGTGCCTAAAGCTG | XM_021098896.1 |
|  | CTAAAGGTGGGAGGATGCTG |  |
| *MUC-2* | GAACGGGGCCATGGTCAG | XM_021082584.1 |
|  | AGCATGACCGAGTCCTCTCT |  |
| *Occludin* | TCGGACTATGCGGAGAGAGT | NM_001163647.2 |
|  | ACGCCTCCAAGTTACCACTG |  |
| *Claudin-1* | CATCTTTGTGGCCACTGTTG | NM_001244539.1 |
|  | CCAGTGAAGAGAGCCTGACC |  |
| *Claudin-2* | TCGGACTATGCGGAGAGAGT | NM_001163647.2 |
|  | ACGCCTCCAAGTTACCACTG |  |
| Genes related to inflammation | | |
| *IL-6* | TGGCTACTGCCTTCCCTACC | AF518322.1 |
|  | CAGAGATTTTGCCGAGGATG |  |
| *IL-1β* | GCCAGTCTTCATTGTTCAGGTTT | NM_214055.1 |
|  | ATCTCTTTGGGGCCATCAGC |  |
| *TNF-α* | GGCCCAAGGACTCAGATCAT | NM_214022.1 |
|  | CTGTCCCTCGGCTTTGACAT |  |
| *iNOS* | GAGCCCAGAGGGCTTTATCA | NM_001143690.1 |
|  | GGGAGTCTGGAGATTTCTTTGCT |  |
| Genes related to Zn metabolism | | |
| *ZIP4* | TGCTGAACTTGGCATCTGGG | XM_021090449.1 |
|  | CGCCACGTAGAGAAAGAGGC |  |
| *ZIP5* | GTTTCCTGTTGTCAGGATGCTC | XM_005655608.3 |
|  | GCCAGCAGATGACTCAATGG |  |
| *ZIP8* | AATGCAAAAGGCTCCACACTCT | XM_021101718.1 |
|  | GCCTCCTGGAGAGCCTGAATG |  |
| *ZIP14* | AGCTCAGTTGAAGTGTGGGG | XM_005657235.3 |
|  | AGCACACGGCTGAAAAATGC |  |
| *MT1* | GCTTGGTCTCACCTGCCTC | NM_001001266.2 |
|  | CTCTTCTTGCAGGAGGTGCAT |  |
| *MT2* | CCATGGATCCCAACTGCTCC | XM_003355808.4 |
|  | GCAGGAGCAGCAGCTTTTCT |  |
| *MT3* | TCTCGACATGGACCCTGAGA | NM_214056.1 |
|  | AGGAGCAGCAGCTCTTCTTG |  |
| *ZNT1* | CTCCAACGGGCTGAAATTGGA | NM_001139470.1 |
|  | CAATTCCACCTGGTCCGGTTC |  |

^1^ *GAPDH*, glyceraldehyde 3-phosphate dehydrogenase; *ZO-1*, zonula occludens-1; *MUC-2*, mucin 2; *IL-6*, interleukin-6; *IL-1β*, interleukin-1β; *TNF-α*, tumor necrosis factor-α; *iNOS*, inducible nitric oxide synthase; *ZIP4*, Zn/iron-regulated transporter-like 4; *ZIP5*, Zn/iron-regulated transporter-like 5; *ZIP8*, Zn/iron-regulated transporter-like 8; *ZIP14*, Zn/iron-regulated transporter-like 14; *MT1*, metallothionein 1; *MT2*, metallothionein 2; *MT3*, metallothionein 3; *ZNT1*, Zn transporter 1.
