# Supplementary material for: Impacts of zinc caproate supplementation on growth performance, intestinal health, anti-inflammatory activity, and Zn homeostasis in weaned piglets challenged with Escherichia coli K88
Source: J Anim Sci Biotechnol. 2025 Mar 14;16:44. doi: 10.1186/s40104-025-01172-2 (PMC11908000; doi:10.1186/s40104-025-01172-2)
Supplement: Supplementary file 2 — Additional file 2. Effects of dietary ZnCA supplementation on organ indexes in piglets. [file 40104_2025_1172_MOESM2_ESM.doc]

Additional file 2. Effects of dietary ZnCA supplementation on organ indexes in piglets

Table S2. Effects of dietary ZnCA supplementation on organ indexes in piglets1

| Indexes | Dietary treatments^2^ | | | | SEM^3^ | P-value |
| --- | --- | --- | --- | --- | --- | --- |
|  | CON | NC | PC | ZnCA |  |  |
| Heart, g/kg | 4.85 | 5.45 | 5.96 | 5.285 | 0.197 | 0.266 |
| Liver, g/kg | 31.84 | 32.50 | 32.51 | 31.82 | 1.10 | 0.99 |
| Spleen, g/kg | 2.28 | 2.19 | 2.13 | 2.44 | 0.08 | 0.62 |
| Kidney, g/kg | 2.77 | 3.03 | 3.00 | 3.09 | 0.058 | 0.243 |

^1^ Data represent the mean of six replicate pens per treatment.

^2^ CON: basal diet + saline solution; NC: basal diet + ETEC K88 challenge; PC: basal diet + 2,500 mg/kg of Zn (ZnO) + ETEC K88 challenge; ZnCA: basal diet + 1,600 mg/kg of Zn (ZnCA) + ETEC K88 challenge.

^3^ SEM: standard error of the mean.
